# Retraction: MicroRNA-493 Suppresses Tumor Growth, Invasion and Metastasis of Lung cancer by Regulating E2F1

**DOI:** 10.1371/journal.pone.0227503

**Published:** 2019-12-31

**Authors:** Yixue Gu, Ye Cheng, Ying Song, Zhijie Zhang, Min Deng, Chengkun Wang, Guopei Zheng, Zhimin He

After publication of this article [1], concerns were raised that there appear to be regions of overlap between the following panels of Figs 3, 5, and 6:

- Fig 3B, 95D and 95D/con panels
- Fig 3B, 95D/miR-493 panel and Fig 5E, H1975/si-E2F1 panel
- Fig 5D, 95D/si-E2F1, 95D/miR-493+E2F1, and Fig 5E, H1975/miR-493+E2F1
- ERK data in Fig 6A lanes 1, 2, and Fig 6C lanes 3, 4
- ERK data in Fig 6A, lanes 2, 3, 4 and Fig 6B, lanes 1, 2, 3

In addition, there appear to be vertical discontinuities in the following western blot panels:

- Fig 4E, E2F1 blot, after lane 3
- Fig 6A, p-ERK blot, before lane 3
- Fig 6B, p-Akt blot, before lane 2
- Fig 6B, p-ERK blot, before lane 3
- Fig 6B, p-GSK3β blot, before lane 5
- Fig 6C, p-Akt blot, before lane 3
- Fig 6C, p-ERK blot, after lane 1

The underlying data for the study were not included with the article, although the Data Availability statement notes, “All relevant data are within the paper and its Supporting Information files.”

The authors noted that duplications in Figs 3B, 5D, and 5E resulted from figure preparation errors, and that data from the Fig 3 experiments were errantly reported in Fig 5. They provided replacement data for Figs 5D and 5E, along with several original images for Fig 3; quantitative data for Fig 3A data were not provided. Notably, 7 out of 10 images provided for the H1975/miR-493 experiment in Fig 3B indicated results similar to the H1975/control data, calling into question whether the corresponding figure panel was truly representative of this experiment’s results.

The underlying data for Fig 4E are not available.

The original image data provided in support of Fig 6 indicated that for some panels the labels on the figure did not align with labels on the raw image data, and/or image data had been spliced to rearrange lanes. In addition, according to the raw image data provided, the wrong ERK data were reported in all three panels and different β-actin controls applied to different experiments shown in each figure panel. The data in the figure did not appear to match the raw data provided for Fig 6B p-GSK3β lanes 5, 6 (H1229 data), Fig 6C p-Akt, or Fig 6C p-ERK lane 1. Original data are no longer available to support Fig 6A GSK3β; Fig 6B ERK, E2F1, Akt, p-Akt; and Fig 6C ERK, β-actin.

In discussing these issues, the authors commented that in general western blot experiments included internal reference protein controls (e.g., β-actin), but for technical reasons they obtained target protein and control data using different blots in some cases. Similarly, they noted that in some cases p-ERK and ERK data were obtained using different blots. This aspect of the experimental design calls into question the validity of the control data for the phosphorylation experiments; without sufficient controls the western blot experiments do not adequately support the reported results and conclusions about effects of miR-493 on the Akt and ERK pathways.

Underlying data for most other results reported in the article are available from the authors. The data indicated that G1 and G2 percentages are misreported in Fig 2C for H1975/miR-493, they ought to be reported as 68.05% and 8.0%, respectively.

In addition to the image concerns, there is text overlap between this article [1] and previously published articles.

Specifically, there is overlap with [2] including but not limited to:

- in the Introduction, “been expanded to include a type of non-protein-coding RNA […] crucial roles in tumorigenesis”
- the majority of the text in the Results subsection titled, “E2F1 regulations ERK and PI3K-AKT pathway in 95D and H1975 cells”
- the discussion of the AKT and ERK pathways in the third paragraph of the Discussion

There is also text overlap with [3] in the second paragraph of the Discussion: “is tightly regulated […] aberrant growth of neoplastic cells.”

In light of the number of data reporting concerns, the unavailability of data for several figures of concern, the unresolved issues involving Figs 4 and 6, and the text overlap, which call into question the overall reliability of the reported results and conclusions, the *PLOS ONE* Editors retract this article. The authors apologize for the issues with this article and report that they are conducting additional experiments to examine the reliability of the results in question.

YG and ZH agreed with the retraction. YC, YS, ZZ, MD, CW, and GZ did not reply or could not be reached.
